# FTA-LAMP based biosensor for a rapid in-field detection of *Globodera pallida*—the pale potato cyst nematode

**DOI:** 10.3389/fbioe.2024.1337879

**Published:** 2024-01-18

**Authors:** Maria João Camacho, Débora C. Albuquerque, Maria L. Inácio, Verónica C. Martins, Manuel Mota, Paulo P. Freitas, Eugénia de Andrade

**Affiliations:** ^1^ INIAV—National Institute for Agriculture and Veterinary Research, Oeiras, Portugal; ^2^ NemaLab/ MED—Mediterranean Institute for Agriculture, Environment and Development, Institute for Advanced Studies and Research, University of Évora, Évora, Portugal; ^3^ INESC Microsistemas e Nanotecnologias, Lisbon, Portugal; ^4^ IST—Instituto Superior Técnico, University of Lisbon, Lisbon, Portugal; ^5^ GREEN-IT Bioresources for Sustainability, ITQB NOVA, Oeiras, Portugal; ^6^ INL—International Iberian Nanotechnology Laboratory, Braga, Portugal

**Keywords:** LAMP, magnetoresistive biochips, PCN, FTA-card ®, Lab-on-a-chip

## Abstract

The combination of a sensitive and specific magnetoresistive sensing device with an easy DNA extraction method and a rapid isothermal amplification is presented here targeting the on-site detection of *Globodera pallida*, a potato endoparasitic nematode. FTA-cards were used for DNA extraction, LAMP was the method developed for DNA amplification and a nanoparticle functionalized magnetic-biosensor was used for the detection. The combinatorial effect of these three emerging technologies has the capacity to detect *G. pallida* with a detection limit of one juvenile, even when mixed with other related species. This combined system is far more interesting than what a single technology can provide. Magnetic biosensors can be combined with any DNA extraction protocol and LAMP forming a new solution to target *G. pallida*. The probe designed in this study consistently distinguished *G. pallida* (∆V_ac_^binding^/V_ac_^sensor^ above 1%) from other cyst nematodes (∆V_ac_^binding^/V_ac_^sensor^ below 1%). It was confirmed that DNA either extracted with FTA-cards or Lab extraction Kit was of enough quantity and quality to detect *G. pallida* whenever present (alone or in mixed samples), ensuring probe specificity and sensitivity. This work provides insights for a new strategy to construct advanced devices for pathogens in-field diagnostics. LAMP runs separately but can be easily integrated into a single device.

## 1 Introduction

Sensors are instruments capable of gauging a physical signal and coverting it into an easy-to-read electrical signal. Within the realm of agricultural industry, different types of sensors are used as part of crop management to foster sustainability and enhance crop productivity—a concept referred to as precision agriculture (PA).

These agricultural sensors play a central role in collecting data during crop growth cycle, from seed-plot to harvest, providing farmers a large amount of information to optimize their decision-making process. They have the capability to measure a wide range of parameters, including but not limited to air temperature, atmospheric pressure, rainfall, wind direction, solar radiation, soil moisture, temperature, nutrient content, electric conductivity and pH at different depths, light reflectance frequencies, carbon dioxide concentrations and other volatile substances. These measurements are used to monitoring crops health (Bogue, 2017; MacDougall et al., 2022).

Sensors are designed to detect physico-chemical parameters. For crop pests/diseases, are necessary biosensors, which combine a transducer with a biological receptor to achieve sensitive and selective detection of a range of analytes. When it comes to detecting crop pests and diseases, there are an increasing number of biosensors available (Mahlein, 2016). Advances in biosensing, information technologies, and nanotechnologies are opening up new opportunities for PA. Optical and thermal sensors are well-suited for detecting patches in the field afflicted by soilborne pathogens during crop production. However, these technologies encounter challenges in accurately differentiating between symptoms caused by different plant pests or diseases and damages resulting from abiotic stresses (Mahlein, 2016; Shao et al., 2023). PA relies on specialized equipment and software to capture data and to infer the expertise of various scientific domains such as, plant health and informatics. There is still room for improvement in all these scientific areas, but a critical aspect in enhancing plant health management is the integration of the different technologies, allowing farmers to swiftly and precisely detect crop pests and diseases. This is where microfluidic sensing devices come into play. Loop Isothermal Amplification (LAMP)-based microdevices have been developed for plant pathogens detection (Fu et al., 2021; Sivakumar et al., 2021) and fully integrated microfluidic devices, comprising DNA extraction, amplification, and the detection of different plant pathogens, were already developed by Loo *et al.* (2017), Wu *et al.* (2021) and Das *et al.* (2022).

In this work, a microfluidic-based portable magnetoresistive (MR) device was adapted, that has been previously developed for detecting different pathogens affecting human and animal health (Martins et al., 2009; Viveiros et al., 2020; Albuquerque et al., 2022), to plant parasitic nematodes detection, namely, potato cyst nematodes (PCN).

The device was specifically modified to detect the internal transcribed spacer (ITS) rDNA region of *Globodera pallida*, which was used as a model organism and is distinct from other related species. The portable analytical platform (Germano and Martins, 2009), comprising the electronic reader and biochips, was a collaborative effort between INESC-MN (Instituto de Engenharia de Sistemas e Computadores—Microsistemas e Nanotecnologias, Lisbon, Portugal) and INESC-ID (Investigação e Desenvolvimento, Lisbon, Portugal). The biochips microfabrication, characterization, and encapsulation on chip carriers was performed in the clean room at INESC-MN. The MR biochip includes six discrete sensing areas, framed by gold squares, with each area containing five MR-based sensors (Spin valves - SV), resulting in a total of 30 active sensors per biochip (Martins et al., 2009). Sensor functionalization involves the immobilization of an oligoprobe with a thiol group, facilitating strong chemisorption onto the gold-pads of the sensing sites (Martins et al., 2009). This biologically active layer on the top of each individual sensor enables the hybridization of the biotinylated target sequence, through specific interactions between complementary sequences of the probe and the target LAMP amplified sequence. Magnetic labelling is achieved by flowing the streptavidin-modified magnetic nanoparticles (MNPs) over the biotinylated target molecules that have been previously immobilized on the sensors, within an U-shaped microfluidic PDMS-channel. Following the removal of unbound entities, the observed variation in the sensor’s electrical resistance (baseline signal - MNPs signal = V_ac_^sensor^ - V_ac_^particles^ = ∆V_ac_^binding^ signal) corresponds to the amount of hybridized target molecules.

Previously, this technique has been successfully demonstrated for the detection of *Globodera pallida* using a laboratory DNA extraction kit and asymmetric PCR, as documented by Camacho *et al.* (2023). However, in order to enhance field-testing conditions and streamline the process, reducing the time and sample preparation requirements, in this study it was opted to use the Whatman Flinder Technology Associates (FTA) cards (Whatman, Maidstone, United Kingdom) rather than the DNeasy Blood and Tissue Kit (Qiagen, Hilden, Germany) to extract DNA. FTA cards are composed of cellulose and include patented chemicals that burst cells, denature proteins, and adsorb nucleic acids, allowing for long-term preservation at room temperature while maintaining quality needed for molecular assays; and have previously been demonstrated to be suitable and effective for plant parasitic nematodes DNA extraction (Marek et al., 2014).

Additionally, a LAMP assay was developed to amplify a DNA fragment of *G. pallida* with the appropriate length to hybridize with newly designed probe.

LAMP was developed by Notomi et al. (2000) as an alternative method for rapid and accurate nucleic acid amplification. It is advantageous because it operates at a constant temperature (typically between 60°C and 65°C), yielding large amounts of LAMP products within a short timeframe (from 20 to 60 min). Therefore, LAMP only requires a straightforward heating device keeping a stable temperature, which is readily compatible with a microfluidic system (Wong et al., 2017; Das et al., 2022). Previous studies have confirmed the suitability of LAMP assays for detecting *G. pallida* (Camacho et al., 2021; Bairwa et al., 2023), the chosen model organism for evaluation in this work.

*Globodera pallida* and *G. rostochiensis*, commonly referred to as PCN, are prevalent nematode species in potato crops. These microscopic, worm-like endoparasites feed on potato roots, deteriorating the quality of tubers, causing a significant reduction in yield, increasing the overall costs of production, and imposing trade restrictions. Both species are worldwide distributed, with *G. rostochiensis* historically having a wider range compared to the more limited distribution of *G. pallida*. Managing *G. pallida* is particularly challenging due to the limited availability of attractive potato cultivars resistant/tolerant to this nematode, whereas several cultivars exhibit high tolerance to *G. rostochiensis*. However, there is a shifting balance between both species driven by the pressure of selection resulting from current nematode management practices, leading to the dominance of *G. pallida* in some countries (Minnis et al., 2002; Camacho et al., 2020).

Owing the detrimental effects of *G. pallida* on potato crops and the difficulties associated with its management, the development of a portable device for a field-specific early detection is crucial to prevent its dispersion. Microfluidics biochips combined with FTA cards and LAMP are a promising solution for on-site detection, enabling the implementation of effective integrated pest management strategies. This work represents a step towards a fully integrated device for rapid in-field crop pest and disease detections.

## 2 Materials and methods

### 2.1 LAMP primers and probe design

The ITS rDNA region used to design the specific probe and LAMP primers for detecting *G. pallida* were acquired from a previous study envisaging to develop a LAMP assay (Camacho et al., 2020; Camacho et al., 2021). The primers, including F3 and FIP as forward primers, and b-B3 and b-BIP (biotinylated on the 5’ end) as reverse primers, were designed to amplify a 172 bp biotinylated LAMP product. The detection of this product involved hybridization with a specific probe that has been previously immobilized on the chip. The design of the DNA probe followed the criteria outlined in Table 1.

**TABLE 1:** List of criteria for probe design (Viveiros et al., 2020).

Criteria	Functional requirements
Size	≈20 and ≈45 bases
Position (PPS)	100–75 and 75–50
Modification	Thiol group in the 5′end
Spacer	15-T bases in the 5′end
G-C content	40%–60% (recommended)
Max. surface-proximal tails	200 bases (shorter possible)
Max overhanging end	Depends on the amplicon size
Starting/Ending bases	G or C (recommended)
Stretches of a single base	No more than 4 in a row
ΔG value of any self-dimers, hairpins and heterodimers	> −9.0 kcal/mol
Heterodimers	Less than 5 bases in a row
Homologies to non-target	Less than 50%

Key characteristics of the primers and probes, such as guanine and cytosine (GC) content, melting temperature (Tm) and change in free energy of hybridization (∆G) were computed using the Integrated DNA Technologies Oligo Analyzer (RRID:SCR_001363). Furthermore, a probe sequence unrelated with any target sequence was used as a negative internal control to correct the positive signals. The primers and probes properties are resumed in Table 2.

**TABLE 2:** Sequence, size, GC content, and melting temperature (Tm) and change in free energy of hybridization (∆G) of LAMP primers designed based on the ITS-rDNA of *Globodera pallida*, the probe specifically designed to target *Globodera pallida* and the negative control probe.

Primers/Probes	Sequence (5′-3′)	Size (bp)	GC%	Tm (°C)	∆G (kcal/mol)
F3 - Forward outer primer (Camacho et al., 2021)	ACA CAT GCC CGC TAT GTT	18	50.0	54.7	−35.32
FIP - Forward inner primer	ACA CTC ATG TGC CCA CAG GGT GGG CTG GCA CAT TGA T	37	56.8	71.0	−74.76
b-B3—Reverse outer primer	Biotin-CCC TGT GGG CGT GCC A	16	75.0	62.0	−37.33
b-BIP - Reverse inner primer	Biotin-TGG GGT GTA ACC GAT GTT GGT GAG CGA CCC GAC GAC AA	38	57.9	70.8	−79.27
*G. pallida* probe	Thiol-15T-CAC ATT GAT CAA CAA TGT ATG GAC AG	26	53.8	62.1	−51.85
Chikungunya probe (negative control probe)	Thiol-15T-CGC ATA GCA CCA CGA TTA G	19	52.6	53.4	−36.7

The design of these probes and LAMP primers was carried out at the GMO and Molecular Biology lab of INIAV (Oeiras, Portugal) and synthesis was performed by Eurogentec (Seraing, Belgium).

### 2.2 Biochemical reagents

All the solutions for the assay were meticulously prepared with ultra-pure grade water, and the specifics of this preparation is resumed in Table 3.

**TABLE 3:** Biochemical reagents preparation conditions.

Reagent	Prepared with	pH
TE buffer	KH_2_PO_4_ (0.1 M)	7.4adjusted withHCl (1 M)
	Tris (10 mM)	
	EDTA (1 mM)	
Phosphate buffer (PB)	Na_2_HPO_4_ (0.2 M)	7.2
	NaH_2_PO_4_ (0.2 M)	
PB-Tween20	PB buffer with 0.02% (v/v) of Tween^®^ 20 from Promega (Madison, WI, United States of America)	

The Magnetic Nanoparticles (MNP) employed in the study were nanomag®-D, sourced from Micromod (Rostock, Germany). These MNPs consisted of 75%–80% (w/w) magnetite within a dextran matrix (40 kDa), and the particles had a diameter of 250 nm. They were streptavidin coated and exhibited a magnetic moment of approximately 1.6 x 10^−16^ A.m.^2^ when subjected to a 1.2 kA/m magnetizing field, with a susceptibility of χ∼4. Prior of being used, the MNPs required a tenfold dilution from stock solution.

### 2.3 DNA nematode samples

The samples used by Camacho *et al.* (2023), which included nematode isolates of *G. pallida*, *G. rostochiensis*, various mixtures of *G. pallida* and *G. rostochiensis*, *G. tabacum*, and *Heterodera* sp. (as indicated in Table 4) were obtained from the nematode collection of INIAV Nematology lab (NemaINIAV, Oeiras, Portugal).

**TABLE 4:** Samples from Portugal and Netherlands used for FTA-LAMP based biochip assays.

Species	Isolate	Origin	ng/µL
*G. pallida*	SV-18–14599	Portugal	*5.2*
*G. pallida*	NPPO-NL Pa3 HLB	Netherlands	1.4
*G. pallida* (FTA)	SV-20–1451–8	Portugal	143
1 *Gp*/5 *Gr*	SV-18–14599/SV-18–14598	Portugal	4
1 *Gp*/19 *Gr*	SV-18–14599/SV-18–14598	Portugal	2.2
1 *Gp*/40 *Gr*	SV-18–14599/SV-18–14598	Portugal	5.4
*G. rostochiensis*	SV-18–14598	Portugal	28.2
*G. rostochiensis*	NPPO-NL Ro1 HLB	Netherlands	2.9
*G. tabacum*	NPPO-NL C6876	Netherlands	39.4
*Heterodera* sp	SV-18–10003	Portugal	18.1

*1Gp/5Gr*, *1Gp/19Gr* and *1Gp/40Gr* represents 1 G. pallida juvenile mixed with 5, 19 or 40 *G. rostochiensis* juneniles.

### 2.4 DNA extraction

**FIGURE 1:**
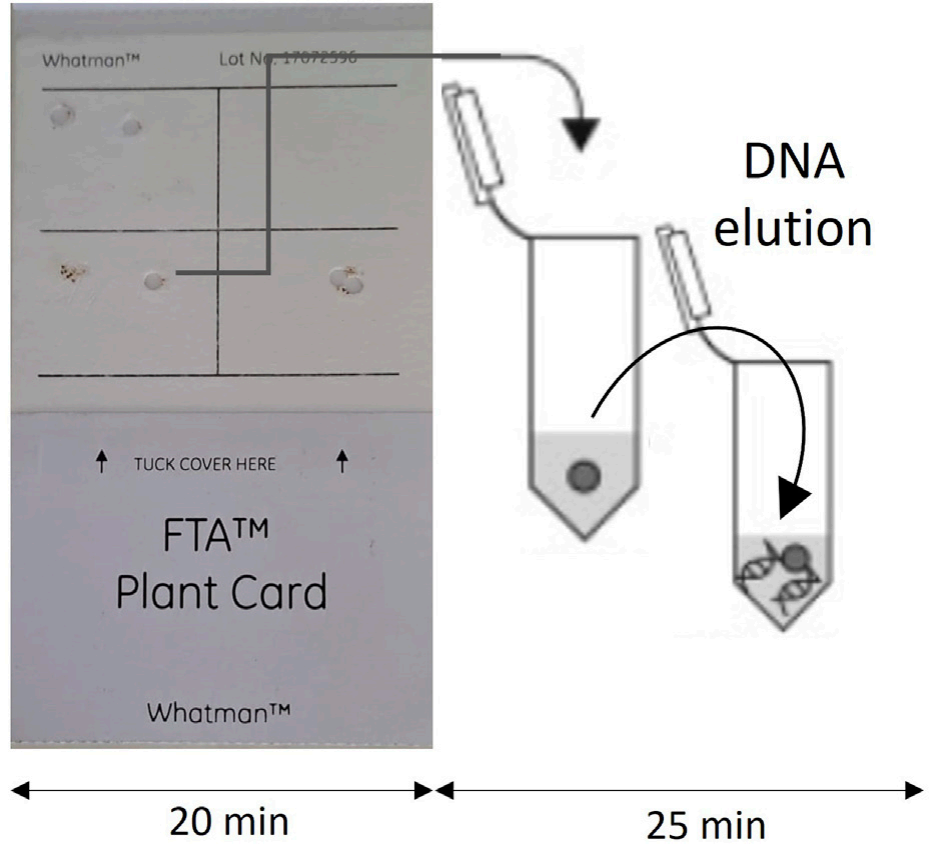
FTA card used for DNA extraction.

Each quadrant of the FTA card was allocated to a single sample. The available cysts were smashed onto the FTA card and let to air-dry for 20 min. At the place where each cyst was smashed (Figure 1), a small disk was punched out and placed in a 1.5 mL tube containing 150 µL of water (DNase and RNase free). These disks were then subjected to incubation in a thermomixer at 70°C for 25 min and subsequently stored at −20°C until needed for further analysis. The total DNA content was quantified using the thermo-NANODROP 2000 spectrophotometer (Thermo Fisher Scientific, Waltham, MA, United States). To compare the efficacy of the DNA extraction method at the lab with FTA cards, the extraction of the other samples DNA was conducted using the DNeasy Blood and Tissue Kit (Qiagen) and following the manufacturer’s instructions. The DNA extracts (Table 4) were directly used for the LAMP reactions without the need for any additional purification step.

### 2.5 DNA amplification

All LAMP reactions were conducted in the B-cube device (Hyris, London, UK) utilizing 16-well cartridges. Each reaction had a final volume of 25 μL, comprising 15 µL of the isothermal master mix ISO-004 (OptiGene, Horsham, UK), along with 0.8 µL of FIP and b-BIP primers (50 µM), 0.15 µL of F3 and b-B3 primers, and 5 µL of the template DNA. The specific isothermal conditions are summarized in Table 5.

**TABLE 5:** LAMP Isothermal conditions.

Amplification temperature, time (min)	Temperature of melting (Heat-Cooling) (°C)
65°C, 60	95°C—75

### 2.6 Detection assays in the biochip platform

**FIGURE 2:**
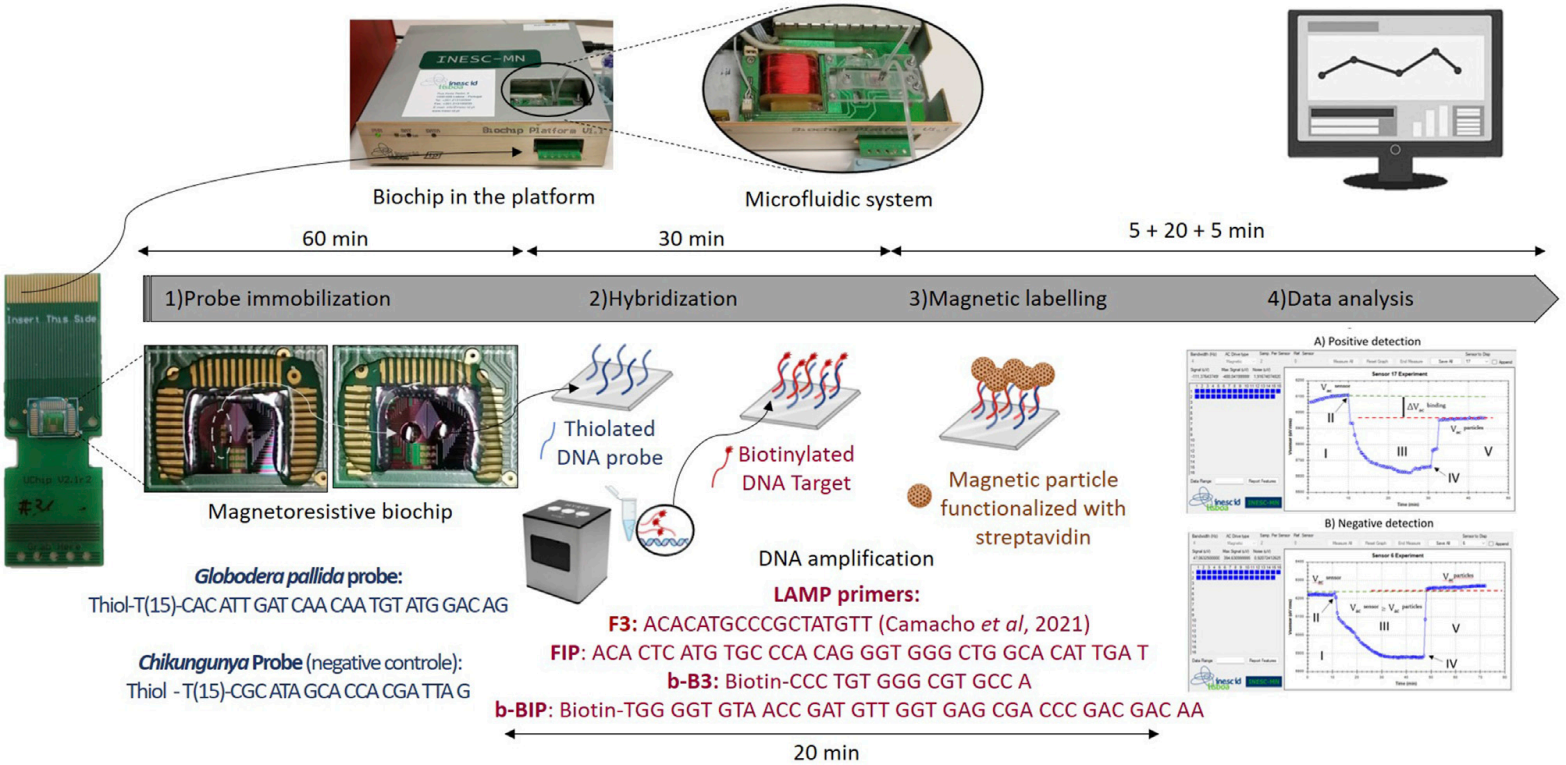
Schematic representation of the main steps involved in a measurement. Step 1) Probe immobilization—Probes are immobilized over the sensing areas (positive probe at the left side, corresponding to sensors 1 to 15—Circled area, and negative probe at the right side, corresponding to sensors 16–30), Step 2) LAMP products hybridization, Step 3) Magnetic labelling of Magnetic nanoparticles through a streptavidin-biotine interaction, and Step 4) Mesurment and data analysis, above is a positive mesurment and below is a negative mesurment.

MR sensor microfabrication is described in the work of Martins *et al.* (2009) and Viveiros *et al.* (2020). The schematic representation of the main steps involved in a positive or negative detection is represented in Figure 2.

#### 2.6.1 Chip functionalization

Before probe immobilization, the biochips underwent a cleaning procedure as described in Viveiros *et al.* (2020).

**FIGURE 3:**
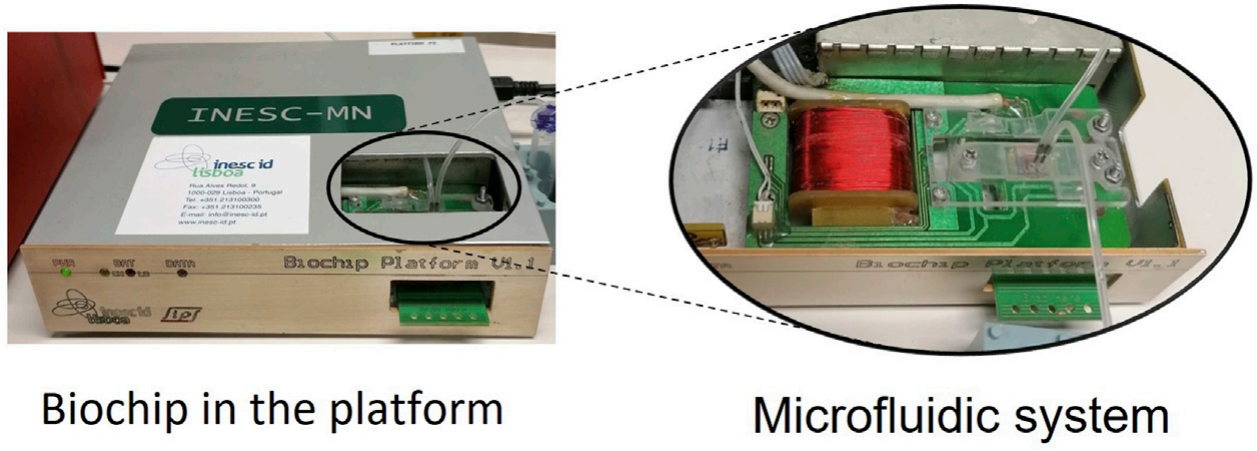
Platform and microfluidic system.

For *G. pallida* detection, the probe was diluted to a concentration of 5 µM in the TE buffer (Table 3), and 1 µL of this solution was spotted on the left side of the sensing area of biochip surface—sensors 1 to 15 (area encircled and zoomed in Figure 2 - Probe immobilization). The same procedure was followed for the negative control probe (the negative probe was used for Shikungunya detections and was spotted on the right side of the sensing area of biochip surface—sensors 16–30). After a 1-h incubation period, the chip was rinsed with PB buffer and then inserted in the platform. The U-shaped PDMS microfluidic system was placed over the sensor to transport the reagents (Figure 3).

#### 2.6.2 Biochip platform measurement

**FIGURE 4:**
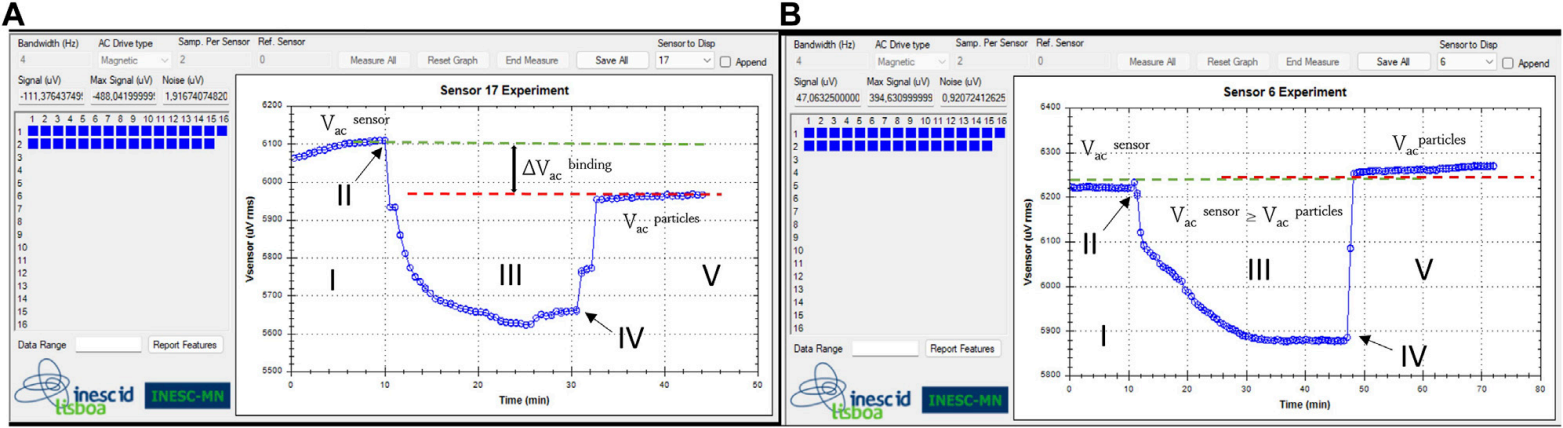
Steps of voltage signal measurements, obtained simultaneously from two different sensors: Step I: Base line signal acquisition (V_ac_^sensor^); Step II: Injection of magnetic particles; Step III: Signal changes due to the presence of magnetic particles over the sensor; Step IV: saturation signal and washing step; Step V: final signal due to the presence of target bound magnetic particles over the sensor (V_ac_^particles^). **(A)** positive detection event: hybridization with a complementary target DNA (*Globodera pallida*) - ending at a lower voltage and **(B)** negative detection event: non-hybridization with a non-target DNA (*G. rostochiensis*) - ending at a higher voltage value.

Initially, the sensors were washed with PB buffer to remove weakly bound probes and establish equilibrium within the system. Subsequently, 10 µL of target LAMP product (previously melted at 90°C for 5 min to denature the DNA) were applied over the sensing sites and allowed to incubate for 30 min. After target-probe hybridization, any unbound target molecules were washed off by rinsing with PB buffer. The measurement with MR sensors started by acquiring the baseline voltage for 5 min (Step I in Figure 4). Then, the magnetic nanoparticles were introduced into the microfluidic system (Step II in Figure 4) and left to incubate over the sensing area for 20 min (Step III in Figure 4). Once the resistance signal of the sensors saturates (Step IV in Figure 4), any unbound particles were washed away within a 5 min timeframe at continuous flow. If the signal stabilizes before 5 min, the wash can be stopped since all the unbound particles were totally washed (Step V in Figure 4). All reagents were loaded at a flow rate of 50 μL/min with the help of a syringe pump (NE-300, NEW ERA, NY, United States). In total, the data acquisition process took approximately 30 min.

#### 2.6.3 Data analysis

The binding signals were determined by calculating the difference between sensor baseline (V_ac_^sensor^) and the signal originated from the MNPs specifically bound to the sensor (V_ac_^particles^). Then, the voltage differential values (∆V_ac_^binding^ signal) were normalized based on the sensor’s baseline and taken as the final output readout signal (∆V_ac_^binding^/V_ac_^sensor^)x100. At the same time, a reference spot (negative control - spotted on the right side of the sensing area of biochip surface), as shown in Figure 2 - Probe immobilization, was established using an unspecific probe (whose target is *Chikungunya* **-** Table 3). The final calculated output signal was the percentage difference of the sensor signals average obtained from positive and negative sensores. This was done to remove the influence of unspecific binding and any potential signal drift. The measurement curves in Figure 4 correspond to the sensors used to detect A) target and B) non-complementary target DNA.

## 3 Results and discussion

### 3.1 LAMP

**FIGURE 5:**
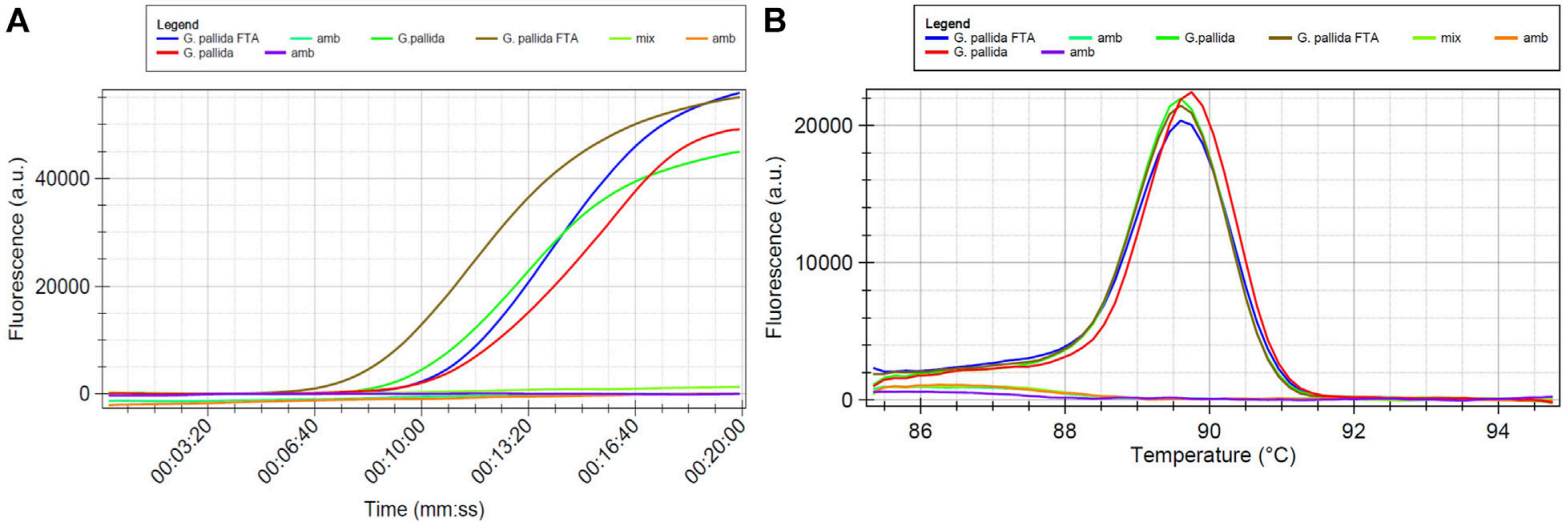
LAMP sensitivity assay using *Globodera pallida* DNA extracted with FTA Cards and with Qiagen kit. Mix and amb samples are non-template control prepared in different working areas of the laboratory **(A)** Isothermal amplification curves and **(B)** derivative of the melting temperature curve.

**FIGURE 6:**
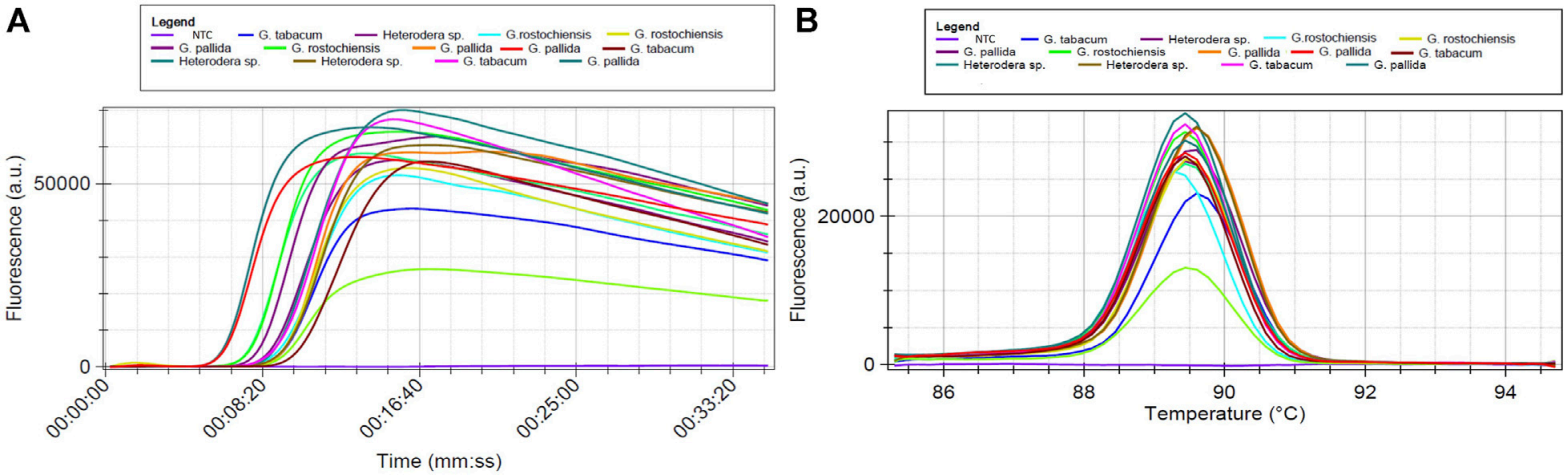
LAMP assay using total DNA of *Globodera pallida, G. rostochiensis, G. tabacum* and *Heterodera* sp. NTC is a non-template control sample: **(A)** Isothermal amplification curves and **(B)** derivative of the melting temperature curves.

**FIGURE 7:**
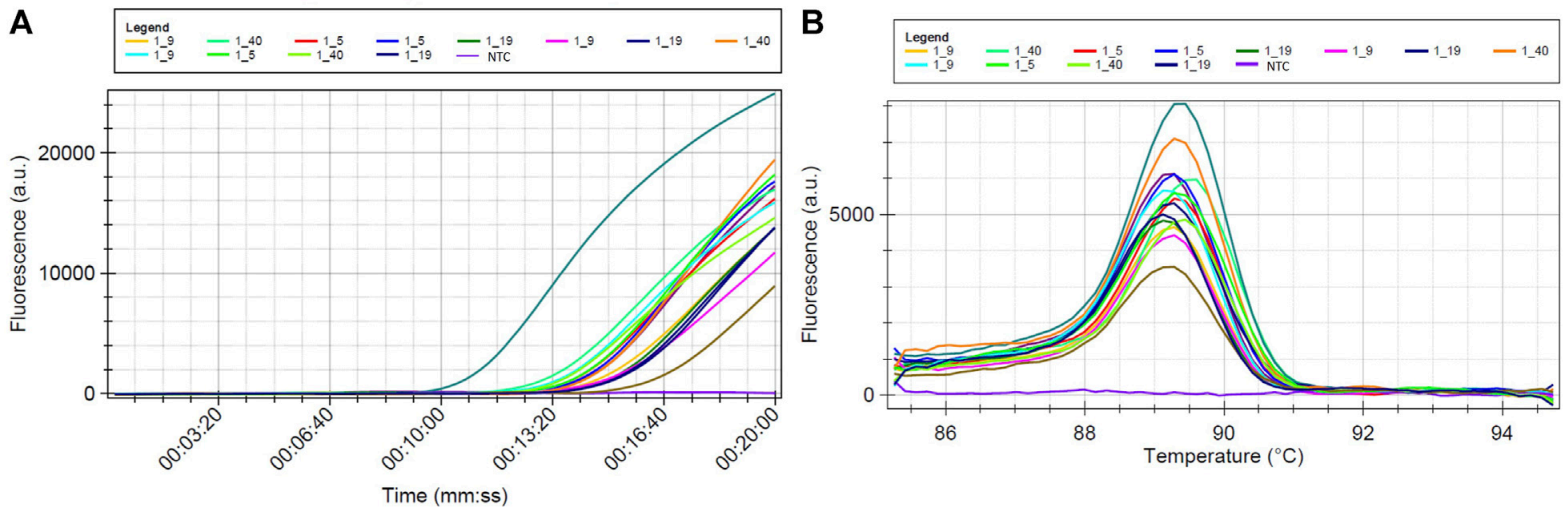
LAMP sensitivity assay using total DNA of pools having different ratios of *Globodera pallida/G. rostochiensis* second stage juveniles (1–5, 1–9, 1–19 and 1–40 represents 1 *G. pallida* juvenile mixed with 5, 9, 19 or 40 *G. rostochiensis* juneniles, NTC is a non-template control sample): **(A)** Isothermal amplification curves and **(B)** derivative of the melting temperature curve.

The DNA samples listed in Table 4 were amplified through LAMP, using the primers indicated in Table 2. These primers were designed based on a region within the ITS-rDNA that remains conserved across various isolates of *G. pallida*, but displays variability among other species. The LAMP amplification products are visually illustrated in Figures 5–7. In all LAMP reactions, the acceptance criterion for a positive result combines a sigmoid amplification curve within 40 min (Figure 5A; Figure 6A; Figure 7A) with a clear pick at the expected temperature on the derivative of the melting temperature curve (Figure 5B; Figure 6B; Figure 7B). In Figure 5, the DNA extraction efficacy with FTA cards was tested. Positive signals were generated after 10 min (Figure 5A) from *G. pallida* DNA extracted with both protocols - FTA cards and Qiagen Kit. No difference between both extrations was observed, demonstrating the efficacy of the FTA cards to field DNA extractions, which has an easier and faster procedure than the Qiagen kit protocol, mainly used for laboratory DNA extraction.

Amplification was detected for all targets, even when the DNA was originated from species other than *G. pallida* (Figure 6). This held true whether the DNA was a mix of different ratios of two species (Figure 7). Despite of having different amplification products, only samples containing DNA of *G. pallida* exhibited successful hybridization with the immobilized probe.

### 3.2 Detection assays in the biochip platform

The detection assays were performed in the MR biochip device with target DNA amplified by means of LAMP (Figures 5–7). All samples were tested with the specific probe for *G. pallida* in the same assay as the negative control probe, which was tested with the specific probe for Chikungunya virus, and used as reference signal. Each sample underwent three or more measurements, corresponding to the detection signal from a range between 12 and 15 sensors in each measurement.

**FIGURE 8:**
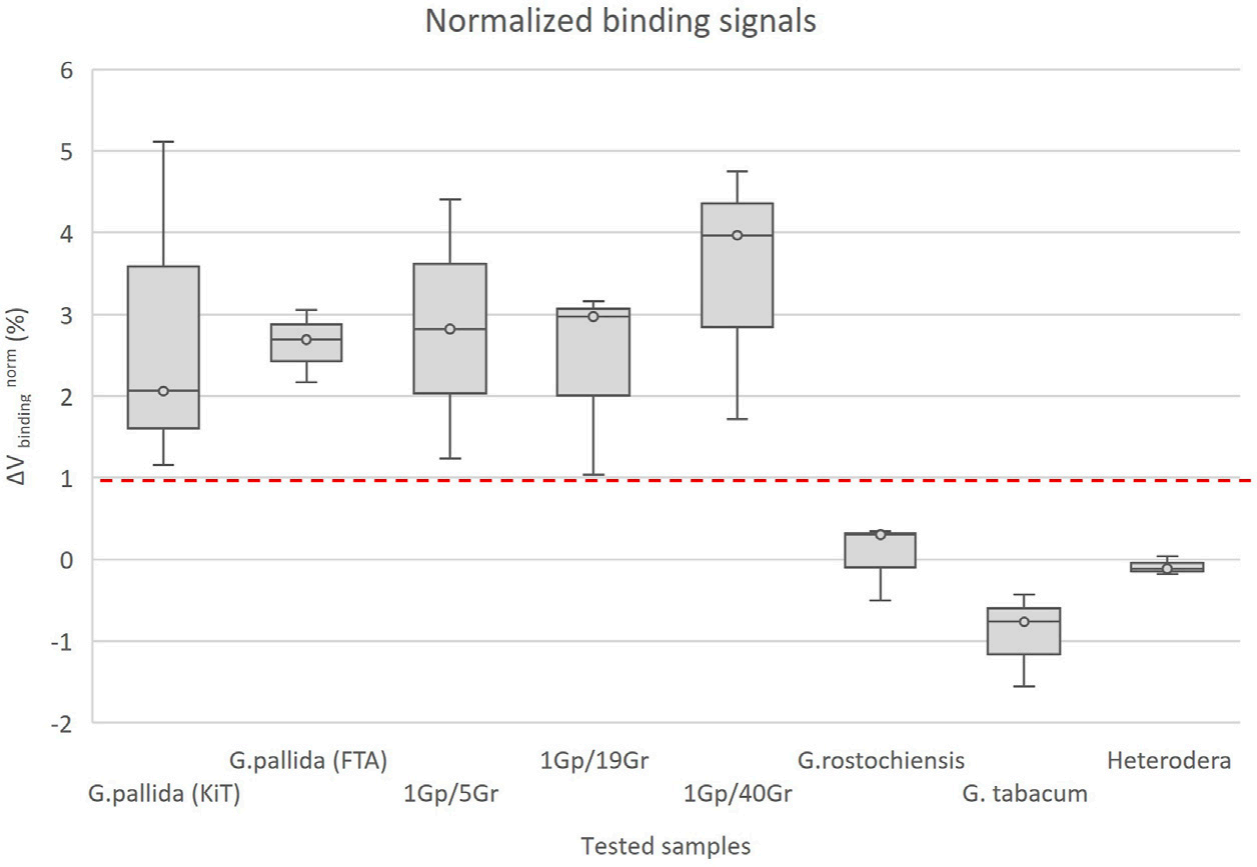
Normalized binding signals obtained from LAMP products of *Globodera pallida*, *G. pallida* extracted with FTA cards, mixed samples of *G. pallida/G. rostochiensis* (1Gp/5Gr, 1Gp/19Gr and 1Gp/40Gr represents 1 *G. pallida* juvenile mixed with 5, 19 and 40 *G. rostochiensis* juveniles - diagnostic sensitivity), *G. rostochiensis*, *G. tabacum* and *Heterodera* sp. (analytical specificity) against the specific probe for *G. pallida* detection. The error bars are standard deviations coming from at least 12 sensors acquired from three measures for each sample. The dashed line represents the threshold. 1%is the value above which a detection signal is considered positive.

At the end of the experiments, the normalized signals acquired from the LAMP products from active and control sensors were compared. The normalized average signals (∆V/Vsensor) acquired for positive sensors covered with *G. pallida* probe or with negative sensors covered Chikungunia probe, after the washing of the unbound MNP on the chip surface at a flow rate of 50 μL/min, presented a clear difference (as shown in Figure 8). In Figure 8, each bar in the graph represents the normalized signal acquired from the LAMP products of *G. pallida*, *G. pallida* extracted from FTA cards, mixed samples, and non-target species (*G. rostochiensis*, *G. tabacum* and *Heterodera* sp.). The threshold value (dashed line) is set at 1%, this value was established as the average ΔV/V from the non-specific signal obtained against a non-complementary target plus its standard deviation of each signal (Viveiros et al., 2020; Albuquerque et al., 2022). It results from the physical behavior of the sensors, and represents the minimal difference in the magnetic field that raises an electrical signal. Signals above this threshold were considered positive detection, signifying a successful match with a complementary target. Conversely, signals below the threshold value were set as negative detection, demonstrating de specificity of the designed probe for *G. pallida*.

The FTA-LAMP-based MR biosensor, funcionalized with a specific oligoprobe (Table 2), showed a remarkable degree of specificity when detecting *G. pallida* LAMP products. This was concluded from the notably free of significant cross-reactivity, enabling the reliable discrimination of this species from other cyst nematodes, including *G. rostochiensis*, *G. tabacum* and *Heterodera* sp. In this analysis, all samples with the target sequence generated detection signals exceeding the 1% (with the average of 2.8% ± 1.3%), while those with non-target sequence produced detection signals below this threshold (with an average of −0.3% ± 0.6%). These findings are aligned with preceding works, where positive detection signals of 1.9% ± 0.8% and 1.8% ± 0.7% were reported, along with negative control results of −0.04% ± 0.4% and 0.4% ± 0.3% (Viveiros et al., 2020; Camacho et al., 2023). The capability to detect *G. pallida* persisted even when working with DNA extracted via FTA Cards or mixed samples containing *G. pallida* and *G. rostochiensis* (with ratios of 1 *G. pallida* juvenile mixed with 5, 19 or 40 *G. rostochiensis* juneniles juvenile: 1/5, 1/19, and 1/40). This underscores a diagnostic sensitivity equivalent to one second stage juvenile (Figure 5).

Developing a mobile biosensor with FTA-LAMP technology application may result in a significant improvement, as it can detect the presence of pathogens directly from environmental samples and has high specificity. FTA-LAMP based microfluidic devices also comes with many advantages, such as easy to operate, expedited and advanced method, palm-sized, high output applicability, and can be applied in the early detection of crop pest and diseases.

## 4 Conclusion

Modern agriculture uses sensor technology to provide accurate and timely data on crop growth, benefiting crop management and yields. The integration of sensors into agriculture systems aligns with the objectives of the European Green Deal, since it offers notable environmental benefits and acknowledges digitization as a tool to enhance productivity by lowering the impact of crop pests and diseases, and enabling an ecological transition, which includes the reduction of pesticides applications. The device used in this work has already been validated for individual detection of bacteria (Fernandes et al., 2014; Barroso et al., 2015; Viveiros et al., 2020), proteins (Albuquerque et al., 2019; Fernandes et al., 2020), nucleic acids (Martins et al., 2009; Dias et al., 2016) and virus (Albuquerque et al., 2022) and can be adapted to field detection of several crop pest and diseases. The FTA-LAMP-based biosensor here demonstrated, is specific for *G. pallida* detection, but can be simultaneously functionalized with species-specific probes for the related species *G. rostochiensis*, *G. tabacum* and *Heterodera* sp., holds significant potential for multiplex detection and for rapid in-field detection or at border phytosanitary inspections.

Although there is still a journey ahead before sensors become commonplace tools in agriculture, the technology underpinning sensors continues to advance, and their use is expected to increase, playing an important role in the future of sustainable agriculture. In this work, a magnetoresistive biochip device was used for the specific detection of *G. pallida* by targeting the ITS-rDNA sequence, and no false positives were observed with closely related species. Our results show that the tested LAMP-FTA based biosensor is exceptionally specific in detecting *G. pallida* even in samples infested with cysts of other *Globodera* species. Thus, the device adapted in this work has proven to simplify and reduce testing time to a complete field detection.

## Data Availability

The raw data supporting the conclusion of this article will be made available by the authors, without undue reservation.
